# MED 117: A dataset of medicinal plants mostly found in Assam with their leaf images, segmented leaf frames and name table

**DOI:** 10.1016/j.dib.2023.108983

**Published:** 2023-02-14

**Authors:** Parismita Sarma, Parvez Aziz Boruah, Rubul Buragohain

**Affiliations:** a Department of Information Technology, Gauhati University, Guwahati, Assam 781014, India; b Department of Botany, Gauhati University, Guwahati, Assam 781014, India

**Keywords:** Medicinal plant, Botanical name, Common name, U-Net segmentation, Watershed segmentation

## Abstract

Medicinal plants are a potential source of income particularly for rural populations in India who rely on these medicinal plants to treat a variety of different diseases through both targeted temporary and daily use. Through this data paper we have given a reference to our collected specimen set where almost 117 (one hundred seventeen) medicinal plant species with their leaf samples are stored. We have used Mendeley platform to store the dataset and visited many medicinal plant gardens situated in Assam to collect them. The dataset consists of raw leaf samples, U-net segmented gray leaf samples and a plant name table. The table includes Botanical name, family of the species, Common name and Assamese name. For segmentation U-net model was used and the resultant U-net segmented gray image frames are uploaded to the database. These segmented samples can be directly used for deep learning model for training and classification. Researchers will be able to use those to build recognition tool for Android or PC based system.

**Specifications Table**

**Table:** 

Subject Area	Horticulture, Herbalism, Digital Image Processing
More Specific Subject Area	Medicinal plant of North East India, Deep learning, Leaf image segmentation and classification
Type of Data	Images of 117 species and total 77,500 image frames and each class contains 179 to 1300 samples. U-Net segmented image of the raw samples. A table with Botanical name, Common Name and Assamese name.
How the Data were acquired	Initial data are taken for at least 30 s duration videos from the source location. We have used a SLR digital camera Nikon, Model:COOLPIX B600 for video recording and a Redmi Note 9 Pro-Max Android phone for capturing photographs of leaves and the plants. The videos were recorded on a white background (i.e.,white paper) which minimizes probable noises that might present. This is an easy and feasible method to segregate the raw videos into frames later on. The videos are recorded from different angles by rotating the camera in clock wise and anti clockwise directions. We tried to take videos of at least three different leaves from each class to avoid texture variability (torn on surface, insect bites or disease infected).
Data format	Raw leaf image data in .jpg format. Analyzed images by using U-Net segmentation in .jpg format. A table of 117 species with serial number, scientific/botanical name, family, common name and Assamese name in .doc format.
Description of data collection	While taking the videos front sides of the leaves were facing to the camera lens. For each sample 180° rotational and 12 inch vertical movements were performed.
Data source location	We have visited a number of medicinal gardens to collect the leaf samples. The most significant sources of medicinal gardens contributed to our work are as follows: (1) Bio-diversity cum Recreation Park, Diphu, Karbi Anglong district of Assam located in altitude 177msl on an swelling background (visited and collected data on 17th October 2021) (2) Medicinal garden situated at North Eastern Development Finance Corporation Ltd. (NEDFi) Research and Development center Khetri- Sonapur, latitude and longitudes of the site are 26.12 and 92.09 at Kamrup District, Assam (visited on 8th November and 30th December,2021), (3) Gauhati University Botanical Garden, located in (latitude and longitudes 26.15, 91.66) Department of Botany, Gauhati university, Guwahati, Assam(visited and collected on 8th January 2021, 8th August 2022), (4) Maharishi Charaka Medicinal Plant Garden situated at Boragaon, (located in 26.11 and 91.68 latitude and longitude) Guwahati, Assam 781,018 (visited and collected on 5th October 2021 and 20th January 2022, 9th August 2022) (5) Kaziranga National Orchid & Biodiversity Park located in 26.58, 93.42 latitude and longitude, situated at 2 km away from the Central Range of Kaziranga National Park, Kohora Chariali Assam visited to collect leaf samples on 13th April 2021 and 19th October 2021.
Data Accessibility:	The data is accessible directly from the following link: MED117_Medicinal Plant Leaf Dataset & Name Table - Mendeley Data The dataset specification is: doi:10.17632/dtvbwrhznz.4. Version 4 Published on 20nd January 2023 Contributors: Parismita Sarma, Parvez Aziz Baruah

## Value of the Data

• Since the ancient times people used to apply parts of different plants from their surroundings as medicine and later this practice contributed to human civilization. Almost all the hilly areas of Assam is famous for various endangered plant species and identification of these plants are really a challenging task. Although most of them are identified yet a huge percentage is still waiting.
• Study coming as result of different research works performed to identify medicinal properties of these plants are now well established and proved. Parts of these plants like fruits, flower, bark, seeds can be used to cure most of the diseases. These plants are now commercially available and treated as nation's source of income.
• Leaf of medicinal plants can be considered as a reliable part for its identification because flowers, fruits and seeds are not found in each and every species but leaves are generally found. Different digital image processing technique can be easily applied on leaf sample on leaf sample is easy because of their structured orientation and features that can be understood by different machine learning segmentation algorithms as well as classification model.
• In this paper we are attaching a set of medicinal plant leaf samples of 117 species and the U-net segmented image set that can be used by other researchers for further classification and recognition. We are also providing their Scientific names, Common names and Assamese names.
• People friendly Android and PC based system/application can be developed for identification of the plants from their leaf.

## 1 Objective

Leaves of plants are generally available throughout the year compared to flowers and fruits that appears seasonally. As such leaves provide a more consistent resource for identification year around. The samples collection and make them suitable to work on digital platform is really difficult. The collected raw samples were in video format and they were partitioned into static images for use in digital image processing algorithms. The dataset mentioned here can help many ways mentioned below..

• Majority of medicinal plant leaves publicly available are stored in single platform. It will contribute in the process of natural resource identification. Recently Government of India is taking interest on cultivation of valued medicinal plants in large scale and thus this small effort may put light on to improve nation's economy.
• This table added to the dataset is a reliable source of information. It was prepared by normal discussion with experts and field executives.
• The raw leaves are preprocessed by using U-Net segmentation technique. These segmented dataset is also made public and accessible through the mentioned link. These samples can be used for image classification and further image processing research.
• There is no dedicated digital or web based system that can recognize a plant from its leaf. With the help of this sample set web based medicinal plant identification system can be built up and it will surely reduce pain of searching and identifying a plant manually.

## 2 Data Description

**Fig. 1:**
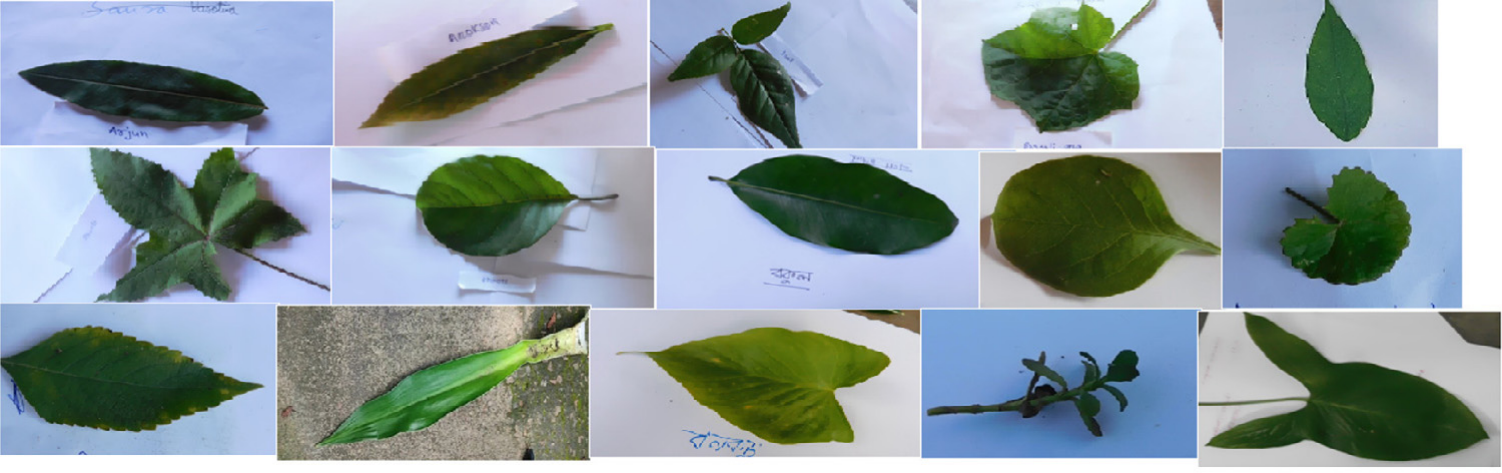
A snapshot of a few medicinal plants.

The videos of the leaves were captured from their front side only. But it was taken from different angles and also vertical rotation of the camera was performed while capturing. Depending upon the rotation we could managed number of frames acquired from every video. Table 1 shows the number of frames collected from each species with their family names. Video data augmentation is used to extend the sample size. In our work we have used a number of transformations to increase the sample size such that our dataset becomes volumeous and ready for deep learning model. Transformations we have used here are cropping, zooming in and out, shifting and rotations. Leaves of a particular plant mostly have same shape, rib and needle orientation but of different sizes. Fig. 1 shows a small set of leaf samples with various shapes and sizes.

**Table 1:** Table of Medicinal plant species with Botanial name, family and sample size.

In this paper we are discussing about segmented dataset also. Model used here to segment the dataset is U-Net segmentation. Users can directly use this dataset for more extensive research work or can use other segmentation method on the raw frames.

In Table 1 all the 117 medicinal plant species are listed. It shows the botanical names, family name inside a big bracket. Total frames present is listed in bold face. The plants are sorted in alphabetic ascending order and read from left to right in each row. Researchers can access these data directly from the web link mentioned in Data Accessibility section.

## 3 Sample Verification

Researchers have used three step verification for sample authentication. We have visited some of the renown medicinal gardens and collected the leaf samples and identified them with the help of the care takers and officials of the respective gardens. Both the leaf samples and snapshots of the plants with name plates (if available) were captured from those sites. We have consulted two renown books written on medicinal plants of North East India. They are:

1. “MEDICINAL PLANTS OF NORTH EAST INDIA” written by professor M. Islam, Department of Life Sciences, Dibrugarh University, Dibrugarh, Assam, Published by Aavishkar, Publishers, Distributors, Jaipur (Raajasthan), India [1].
2. “PLANT FOLK- MEDICINES & MEDICINAL PLANTS OF NORTH EAST INDIA” by Subhan C. Nath, CSIR-North East Institute Of Science & Technology. Jorhat, Published by Dr. P. G. Rao, Director, CSIR-North East Institute Of Science & Technology. Jorhat [2].

Finally from the framed and preprocessed samples a table is prepared which includes Botanical name, Species of the plant, Common name and Assamese name. The table is uploaded in mendeley platform with title “Medicinal Plant Name Table” under the folder “MED 117 Leaf Species”. Superintendent of Botanical Garden, Botany Department, Gauhati University, Guwahati, Assam, India. Dr. Rubul Buragohain, an expert in Angiosperm Taxonomy cross checked the whole dataset and thus the most versatile sample set is prepared.

## 4 Experimental Design, Materials and Methods

### 4.1 Data Acquisition

The sample collection was done in different seasons of the year. In this research extensive field work was carried out at different places of Assam at different time of the year. During winter most of the leaves fall down and we had to wait for them till April. The main sources of sample collection are Bio-diversity cum Recreation Park, Diphu, Karbi Anglong, North Eastern Development Finance Corporation Ltd. (NEDFi), Research and Development center Khetri- Sonapur, Gauhti University Botanical Garden, Department of Botany, Maharishi Charaka Medicinal Plant Garden situated at Boragaon, Guwahati, Kamrup District, Assam, Kaziranga National Orchid & Biodiversity Park, Kaziranga. The species of the collected samples are validated by a taxonomy expert from Botany Department of Gauhati University. In the months of October and December 2021 sample collection, preprocessing and segmentation were completed. Preprocessing and segmentation in second and third phases were completed on April 2022 and August 2022 respectively. More than one video for each plant were taken from more than one location and merged under the same folder.

### 4.2 Pre Processing

The frames are extracted from video footage and saved as images (.jpg format) against each class. These images are the final dataset and can be used for classification using machine learning or deep neural network. The platform used here is OpenCV library functions. We have prepared one hundred seventeen (117) classes of plants and collected total 77,500 raw images. Each plant class contains 179 to 1300 samples.

### 4.3 Segmentation

Image segmentation is the process of breaking down the image into its constituent parts called segments. This is required in image classification because it helps to reduce complexity of the image and prepares for training on the neural network model [3]. All similar picture elements (i.e., pixels) are collected under one group. Thus different groups are formed with similar feature values. Segmentation removes background noises and unwanted regions from the image. We have used here U-Net segmentation algorithms to segment the raw dataset and the Gray segmented dataset is uploaded in the sub folder “Segmented leaf set using UNET segmentation” in mendeley platform.

#### 4.3.1 U-Net Model

**Fig. 2:**
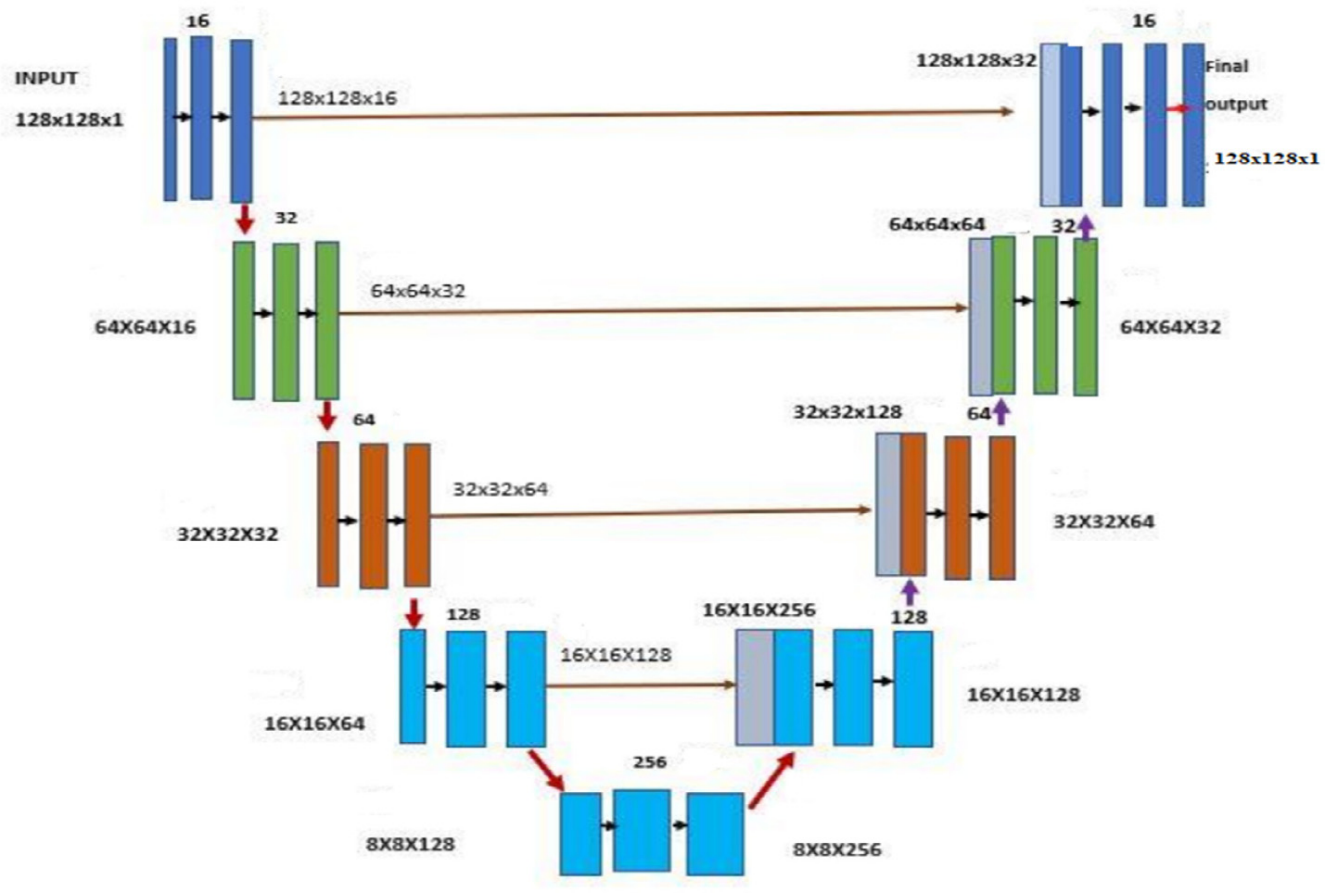
Basic architecture of U-net model.

Originally U-Net was designed for segmentation of biomedical images. This segmentation method helps to focus on area of divergence. In general Convolution Neural Network (CNN) is used for labeling where input is image and output is label. Classification is performed on every pixel, so both input and output image enclose the same size. Fig. 2 shows the basic model for U-Net architecture comprised of a constricting path (decreases with the input size) and an expansive path (increases with its input size). The contracting path is serial blocks of Convolutional network, each block consists of two Convolutions layers, a Rectified Linear Unit (ReLU) activation function and a max pooling layer for down sampling. Every down sampling step is increased by twice the number of feature channels [4].

On the other hand the expansive path consists of an up sampling of the feature map. Then there is a convolution (up-convolution) which helps to reduce the number of feature channels by 50%, next a concatenation with the equivalent cropped feature maps form the contracting path and a Convolutional layer, followed by a ReLU activation function. The target of the expansion path is to semantically map the discriminative features (in lower resolution) that was learnt by the contracting path onto the pixel space (in higher resolution) to get a dense classification. To reduce loss on border pixels in each convolution step cropping is performed. The final layer uses convolution for mapping the feature vectors to the needed number of classes [5].

By using Transposed convolution (De convolution) expansion path up sampling can be achieved. Basically the transposed convolutions is applied in opposite direction of normal transformation. Fig. 2 shows the architecture of our U-Net model where input image size is 128 × 128, 16 bit, down sampling, achieved 256 bits, again up sampling and achieved 16 bits original.

We have taken 1812 images randomly from our leaf dataset. We have taken 10 to 20 samples from each of the leaf classes. These images are generally the segmented images which are achieved by traditional watershed segmentation approach. After performing the segmentation we have converted it to binary images by using thresholding function in OpenCV library. This binary images build our target images during training the U-net model. Table 2 shows a summary representation of our U-Net model that was used to segment the leaf samples.

**Table 2:** Parameters of our U-net (Gray scale) model.

Model	Input specification	Numbers of Training Samples	Train/Val Split	Epoch	Train Accuracy	Val Accuracy	Training Time Taken
U-Net (GrayScale)	(256,256,1)	1816	9:1	40	96.33%	95.49%	1519 s

Among these 117 (one seventeen) species, Asparagus officinalis L.(Wild asparagus), Crinum viviparum (Lam.)(Indian-squill) and Bacopa monnieri (L.) Wettst.(Water hyssop) segmented frames were not clearly understandable. So Asparagus officinalis L.(Wild asparagus) and Crinum viviparum (Lam.)(Indian-squill) were excluded from the segmented dataset. Yet we retained Bacopa monnieri (L.) Wettst (brahmi) in that folder.

Table 3 shows some segmented leaf images of eight medicinal plants which were segmented initially by using U-Net model but the resultant images were not good and clear. So Watershed segmentation method was applied on those resultant images and masking was done on them. Table 3 shows it clearly. The first column holds serial number, second column lists species name, third column images of U-Net segmented leaves of each listed species, fourth column includes images of U-Net + watershed segmented leaf samples.

**Table 3:** Species segmented by U-Net then Watershed segmentation technique.

## Ethics Statement

The research work described here neither involves any human beings nor animals. All the experiments were performed with no harms on human body, animals, birds, insects etc. All the images included in this article were collected by the researchers themselves, no image or species is taken from any electronic media, books, papers, journals etc. We have received fund for carrying out this experiment from ASTEC (Assam Science Technology & Environment Council), Govt of Assam.

## CRediT Author Statement

**Parismita Sarma:** Data Acquisitions, Data Analysis, Conceptualization, Methodology, Writing, Project Administration; **Parvez Aziz Boruah:** Data Acquisitions, Data Curator, Methodology, Software; **Rubul Buragohain:** Investigation, Sample Verification, Data Analysis.

## Declaration of Competing Interest

The authors declare that there is no known competing financial interest or personal relationships which have or could have influenced the research carried out and shown here.

## Data Availability

MED117_Medicinal Plant Leaf Dataset & Name Table (Original data) (Mendeley Data).
